# Consequences of Perceived Personal and Group Discrimination against People with Hearing and Visual Impairments

**DOI:** 10.3390/ijerph18179064

**Published:** 2021-08-27

**Authors:** Daniel Pérez-Garín, Patricia Recio, Fernando Molero

**Affiliations:** 1 Faculty of Psychology, National University for Distance Education (UNED), 28040 Madrid, Spain; dapgarin@psi.uned.es (D.P.-G.); reciop@psi.uned.es (P.R.); 2 Joint Research Institute IMIENS, 28029 Madrid, Spain

**Keywords:** hearing impairment, visual impairment, perceived discrimination, internalized stigma, self-esteem, group identification

## Abstract

The main objective of this study was to examine the consequences of perceived discrimination in people with hearing and visual impairments. Using path analysis, we attempted to validate a multigroup model in which perceived personal discrimination is associated with internalization of stigma, which, in turn, is negatively related to self-esteem; conversely, perceived discrimination against the in-group contributes to enhanced group identification, which promotes the intention to engage in collective action, which, in turn, has beneficial effects on self-esteem. The sample consisted of a total of 200 Spanish-speaking participants, of whom 104 had hearing impairments and 96 had visual impairments. The results showed that the proposed multigroup model fit the data well. For both groups, internalized stigma played a mediating role in the relationship between perceived personal discrimination and self-esteem. However, the pathway from group discrimination to self-esteem was not as clearly supported by the data. The results are interpreted from a psychosocial perspective and may contribute to design interventions aimed at improving the well-being of people with hearing and visual impairments.

## 1. Introduction

People with hearing or visual impairments often suffer from discrimination or “ableism”. In disability studies, the concept of “ableism” refers to a kind of discrimination in favor of able-bodied people involving the notion that “disability may be tolerated but, in the final instance, is inherently negative” [1] (p. 151). This is a concept very similar to that of stigma; in fact, since its very beginning, the literature on stigma has taken account of disability, which is one of the three kinds of stigmas identified by Goffman [2] along with those associated with character traits and identification with a devaluated race, ethnicity, religion, or ideology.

According to Crocker, Major, and Steele [3] (p. 505), “stigmatized individuals possess (or are believed to possess) some attribute, or characteristic, that conveys a social identity that is devalued in some particular context”; having a hearing or visual impairment may constitute such an attribute. According to Fine and Asch [4], many of the difficulties faced by people with disabilities in their daily lives are not directly caused by their disabilities but rather by a disabling environment that builds barriers (both physical and social) that exacerbate exclusion and discrimination. In other words, many of those barriers are, in fact, a product of stigma and not of disability, and society’s failure to acknowledge this makes public and structural stigma worse.

### 1.1. The Stigma of Hearing and Visual Impairments

The literature shows that people with hearing impairments suffer from both public and self-stigma and that this stigma can pose a barrier that prevents them from seeking rehabilitation or using hearing aids [5,6,7,8].

A review of the literature on stigma related to hearing loss and hearing aids among older adults [9,10] found that the most common self-stigmatic behaviors endorsed by elderly people with hearing problems were concealing their hearing difficulties (in various ways, such as pretending to hear what was being said, refraining from making explicit demands that might help facilitate communication, and choosing not to wear hearing aids), denial, and avoiding communicative interactions. This review also shows that having a hearing impairment is a major threat to social identity, threatening the stability of social interaction, and that stigma impedes help-seeking activities. Many people with hearing loss only seek help after a long phase of denial and concealment, during which their stress increases, and their hearing loss worsens.

People with visual impairments also suffer from public stigma and internalized stigma. Some studies have focused on the impact of stigma on the social lives of youths with visual impairments. Research shows that public stigma can take the form of bullying of people with visual impairments [11] and that it can act as a social barrier that hinders the inclusion of youth with visual impairments [12]. Self-stigma, on the other hand, was associated with feelings of loneliness in a sample of Chinese university students with visual impairments; this association was mediated by lower levels of self-acceptance [13]. Similar to what happens to people with hearing impairments, stigma appears to lead people with visual impairments to reject the use of canes and other assistive technologies [14,15]. 

A recent qualitative study [16] assessed perceived discrimination and emotional reactions in a Spanish sample of people with physical disabilities, hearing impairments, and visual impairments. Regarding the latter two groups, which are the target of the present study, the participants with hearing impairments mainly reported encountering barriers in leisure activities and being treated like children, whereas people with visual impairments spoke of a lack of equal opportunities at work (which was also a frequent concern of respondents with hearing impairments), mockery and/or bullying, and overprotection, and also shared the concerns of people with hearing impairments about barriers in leisure activities. Regarding their emotional reactions to discrimination, people with hearing impairments reported feeling anxious, helpless, and frustrated, and people with visual impairments reported feeling anger and self-pity.

### 1.2. Perceptions of Personal Discrimination and Internalization of Stigma

Perceived discrimination can be defined as an individual’s awareness of public stereotypes and discrimination. As shown by two meta-analyses [17,18], perceived discrimination has a detrimental impact on several aspects of the health and well-being of members of different stigmatized groups. Most of the studies included in these meta-analyses, however, refer to racial or sexual discrimination. In this study, we attempted to increase knowledge of the consequences of perceived discrimination experienced by people with sensorial impairments. To the best of our knowledge, no studies have been conducted concerning perceived discrimination in people with hearing impairments, and we were only able to find one about perceived discrimination in older adults with vision impairment [19]. That study showed that older adults with impaired vision are more likely to report perceived discrimination than the general population and that those who reported experiencing discrimination showed higher levels of depressive symptoms and loneliness and lower scores for quality of life and life satisfaction.

Perceived discrimination is not a single construct. The literature shows that the perception of being personally discriminated against for being a member of a devalued group (personal discrimination) and the perception that the in-group as a whole is discriminated against (group discrimination) may have different psychological consequences. Specifically, although perceived individual discrimination is negatively related to self-esteem, this relationship is not so clear in the case of perceived group discrimination. In this case, some studies have found negative associations between group-level perceived discrimination and self-esteem [20] with a sample of people living with HIV, and others have found positive relations between group-level perceived discrimination and self-esteem, particularly in groups based on racial identity [21,22]. One of the objectives of this study is to analyze the relationships between group-based discrimination and self-esteem in the case of people with visual impairments. 

One of the potentially most negative consequences of individual discrimination is the internalization of stigma. Internalized stigma, or self-stigma, is a person’s acceptance of stigma as a part of their value system and self-concept [23,24]. Internalized stigma has mainly been studied in people with mental illness and in people with HIV, finding in both cases very harmful consequences in terms of feelings of blame, anxiety or hopelessness, self-esteem, and self-efficacy [25,26]. In a study by Molero et al. [27], internalized stigma was found to play a mediating role in the association between perceived personal discrimination and self-esteem in a sample of people with physical disabilities.

### 1.3. Perceived Group Discrimination, Group Identification, and Collective Action Intention

An explanation for why perceived group discrimination sometimes seems to have a positive effect on self-esteem is proposed by the rejection-identification model (RIM) [28], which underlines the mediating role of group identification. According to the RIM, the perception of group-based discrimination increases in-group identification, which, in turn, prevents some of the negative effects of discrimination. In other words, group identification plays a mediating role between perceived group discrimination and its negative outcomes.

Evidence supporting the RIM has been found in studies examining the effect of discrimination in a variety of social groups such as women [29], older adults [30], and Black and Latino Americans [28,31]. Other studies, however, provide only partial support for the model [21,22,32] or even no support at all [33,34]. Moreover, a study conducted with people with disabilities shows that the perception of a stigma against the in-group predicts not only group identification, but also disability pride, which in turn predicts higher levels of self-esteem [35].

Other studies have tested the role of group identification as a moderator of the impact of discrimination on well-being. A review by Schmitt et al. [18] found a variety of results in this respect. In 46% of the samples included in the review, no significant moderating effects were found; in 38%, there was at least one significant buffering effect; in 13%, at least one exacerbating effect was found; and one study found both a buffering and an exacerbating effect. The disparity of these results makes it necessary to study the stigmatized identity of two groups that have scarcely been studied from the perspective of stigma: people with hearing impairments and people with visual impairments.

Interestingly, however, the existence of a deaf identity and even a deaf culture is well documented, and a strong deaf identity has been shown to have a positive association with self-esteem [36,37]. There is also research documenting the fact that people with visual impairments can develop a positive blind identity [38].

In this study, we assessed group identification through a Spanish adaptation [39] of the classic scale of Mael and Ashforth’s Organizational Identification Questionnaire [40]. This questionnaire assesses to what extent the individual feels identified or shares some characteristics with other members of their group (in this case, people with visual or hearing impairments). 

Although the relationship between group identification and well-being has not always been supported, the relationship between group identification and collective action intention is well established in the literature [41]. In fact, identification with the in-group is a precondition for acting on behalf of the group, either to enhance its status [42,43,44] or to improve its material conditions (e.g., improving the accessibility of different types of media, such as websites or TV channels for people with sensory impairments). History provides many examples of groups that aimed to improve their position by means of collective action (e.g., feminism, the gay movement, striking miners in the UK). Collective action includes not only militant forms of intergroup action (e.g., revolts, strikes), but also more moderate forms (e.g., participation in social movements, signing a petition).

Regarding stigmatized groups, there are several studies showing that in-group identification is related to collective action intention in people with HIV [20], lesbians and gay men [45], and people with mental illness [46]. However, in these studies, the effect of collective action intention on well-being was not so clear. It was significant in the case of people with HIV, but non-significant in the case of lesbians and gay men, and mixed (collective action intention was related positively both to positive and negative affect) in the case of people with mental illness.

A previous study explored the relationship between group discrimination, collective action intention, and self-esteem in a sample of people with physical disabilities [27]. To the best of our knowledge, however, the relationships between these variables have not yet been tested in two other groups of people with sensorial disabilities: people with hearing impairments and people with visual impairments.

### 1.4. The Present Study

**Figure 1:**
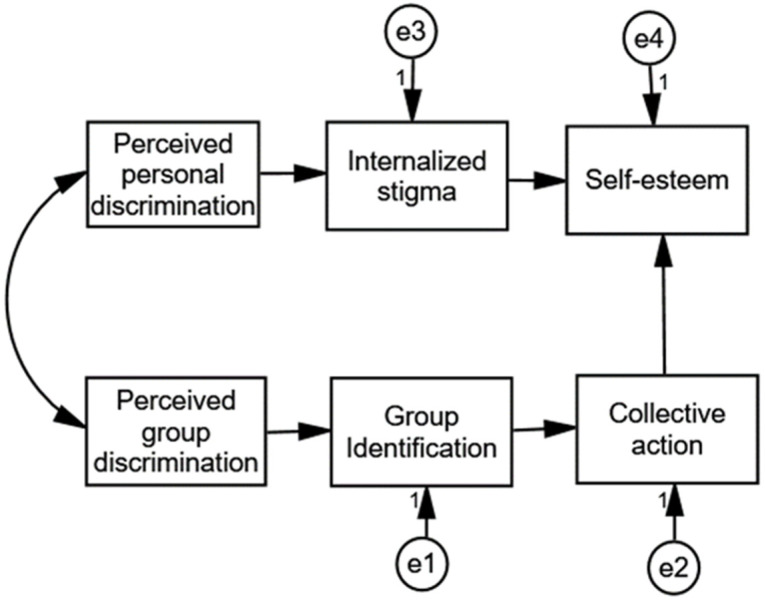
Mediating model proposed for people with hearing and visual impairments.

The main objective of this study was to examine the consequences of perceived discrimination in people with hearing and visual impairments. Using path analysis, we attempted to validate a multigroup model in which individual perceived discrimination is associated with the internalization of stigma, which, in turn, is negatively related to self-esteem; conversely, perceived discrimination against the in-group contributes to an enhanced group identification, which promotes the collective action intention, which, in turn, has beneficial effects on self-esteem. A model similar to the one proposed in this study (see Figure 1) was previously tested for people with physical disabilities, and it showed a good fit for that group [27]. The aim of this study was to assess whether the model also provides a good fit for people with visual impairments and people with hearing impairments. This study is novel not only because it tests this model in two groups on whom such an analysis has not been conducted before, but also because it tests the fit of the model for both groups simultaneously through a multigroup path analysis.

## 2. Materials and Methods

### 2.1. Participants

The sample consisted of a total of 200 Spanish-speaking participants (54% women and 46% men), ranging from 17 to 89 years (M = 45.16; SD = 12.87). Of this sample, 104 had hearing impairments, whereas 96 had visual impairments.

Regarding the degree of disability, as measured and acknowledged by the Spanish authorities, 61.5% of the participants reported a disability percentage between 33% and 65% (which grants them a Disability Certificate, giving them access to certain benefits, rights, and services), and 38.5% reported a disability percentage greater than 65% (which means they also qualify to receive a non-contributory pension).

As for their educational level, 44% of them reported having received secondary education and/or vocational training, 38% received higher education, 17.5% received primary education, and the remaining 0.5% reported having no formal education.

### 2.2. Measures

Multidimensional Perceived Discrimination Scale [47]. This is a 20-item instrument that measures the perception of discrimination of members of stigmatized groups. Although the original authors proposed a four-factor solution (Blatant Group Discrimination, Subtle Group Discrimination, Blatant Personal Discrimination, and Subtle Personal Discrimination), in this study, like Pérez-Garín et al. [46], we grouped the four factors into two, as this served the purpose of this study better, resulting in the following two factors: Group Discrimination and Personal Discrimination. Perceived group discrimination measures the extent to which the respondent perceives discrimination toward their group as a whole, whereas perceived individual discrimination is the extent to which the respondent perceives themselves as having been personally discriminated against. The instrument uses a 4-point Likert response scale, with responses ranging from 1 (do not agree at all) to 4 (agree completely), with higher scores indicating higher levels of perceived discrimination. Both subscales showed good internal consistency (Cronbach’s alpha = 0.89 and 0.91 for Group Discrimination and Personal Discrimination, respectively).

The Stigma Scale for Chronic Illness 9-Item Version (SSCI-9). We used the Spanish adaptation of the Internalized Stigma subscale of the SSCI [48], which showed good psychometric properties in a sample of people with different types of disabilities [49]. The SSCI-9 uses a 4-point Likert response scale to assess frequency, ranging from 1 (never or almost never) to 4 (always or almost always). The alpha coefficient for this scale was 0.88 in our sample. Sample items are ““When I talk about people with visual disabilities I usually say ‘we’ rather than ‘they’”, or “When someone criticizes people with visual disabilities, it feels like a personal insult”.

Group Identification. This variable was measured using a modified Spanish version [39] of Mael and Ashforth’s Organizational Identification Questionnaire [40]. The respondents were asked to answer on a 4-point Likert scale, indicating the degree to which they agreed with the statements presented. The instrument showed good reliability in our sample (Cronbach’s alpha = 0.82).

Collective action intention. This was measured with four items in which the respondents were asked how effective they perceived collective action to be and if they intended to engage in it [46]. Sample items are “Collective action is a good way to defend the rights of people with disabilities” or “I am willing to participate in collective actions to support the rights of people with disabilities.” The participants were requested to respond on a Likert scale ranging from 1 (do not agree at all) to 4 (agree completely), with higher scores indicating that the respondent believed that collective action was useful and was willing to participate in it. The internal consistency of the scale was good (α = 0.84).

Self-esteem was measured by the Rosenberg Self-Esteem Scale [50], using the Spanish-language version by Expósito and Moya [51], which is composed of 10 items. The scale was answered on a 4-point Likert scale, ranging from 1 (do not agree at all) to 4 (agree completely). Previous studies have shown that this Spanish version has good psychometric properties [52], this was also the case for the present sample (Cronbach’s alpha = 0.83).

### 2.3. Procedure

The participants were recruited online by social work students at UNED, who voluntarily searched for people with hearing or visual impairments. They explained to the participants the goal of the study, the method that would be used, and the time required to complete the various scales. The respondents completed the consent form and read the first page informing them about the aims of the study. Then filled out an online self-administered questionnaire. The questionnaire answered by the participants with visual impairments had specific adaptations to ensure the accessibility. Anonymity and confidentiality were guaranteed, and the participants were informed that they could withdraw from the study at any time. Completing the questionnaire took approximately 30 min. Questionnaire data were collected for a period of 3 months.

The study was approved by the University Ethics Committee and was performed in accordance with the ethical standards of the Declaration of Helsinki. 

### 2.4. Data Analyses

The data were analyzed using the statistical software package SPSS 25 for descriptive and correlational analysis and then using AMOS 25 for path analysis to test the hypothesized mediating effects and simultaneously explain the interaction between the main variables [53]. As recommended by Kline [54], we considered various indices to assess the adequacy of the model; for acceptable fit: 2/df < 3, CFI > 0.90, NFI > 0.90, and RMSEA < 0.08; and for excellent fit: 2/df < 2, CFI > 0.95, NFI > 0.95, and RMSEA < 0.06. We tested a multigroup model considering the two disabilities. The mediation effects were analyzed for the two subgroups using the bootstrapping method (10,000 replications) with 95% bias-corrected confidence intervals. A requirement for performing a multigroup model is that the model presents a good fit for each of the groups separately, so we tested the fit for each of the groups separately first. In addition, the chi-square difference test (Δχ2) was used to determine whether there was any cross-group invariance when comparing two nested models: the unconstrained model (without constraints specified) and the constrained model (wherein the parameters were constrained equally across the subgroups). The significant values indicate that the less restricted model should be accepted, following the recommendation by Cheung and Rensvold [55].

## 3. Results

### 3.1. Descriptive Analyses and Correlations

Table 1 and Table 2 present the descriptive statistics and correlations of all the variables in the study for people with hearing impairments and people with visual impairments, respectively. The means for both perceived personal and group discrimination were higher for people with hearing impairments than for people with visual impairments (2.38 and 2.59, respectively, for people with hearing impairments and 2.03 and 2.27, respectively, for people with visual impairments). The mean for internalized stigma was higher for people with hearing impairments than for people with visual impairments (2.20 and 1.96, respectively), whereas self-esteem was lower (3.15 and 3.33, respectively). According to the results of the t-tests, all of the differences were significant (with *p* < 0.01 for both measures of perceived discrimination and for internalized stigma, and *p* < 0.05 for self-esteem).

On the one hand, the pattern of correlations for people with hearing impairments was, in general, in accordance with our expectations (perceived personal discrimination was negatively associated with self-esteem and positively associated with internalized stigma, whereas group discrimination was positively associated with group identification). However, neither the correlation between group identification and self-esteem nor the correlation between collective action and self-esteem were significant.

On the other hand, in the sample of people with visual impairments, the correlation between personal discrimination and self-esteem was not significant, but collective action was significantly associated with self-esteem.

### 3.2. Model Testing and Multiple-Group Structural Equation Model Analysis

The hypothetical model was first tested between each group separately and demonstrated an acceptable fit to our data for both the hearing impairments model (χ2 (9) = 9.13, *p* = 0.425; CFI = 0.999; NFI = 0.960; RMSEA = 0.012) and the visually impaired people model (χ2 (9) = 8.98, *p* = 0.724; CFI = 1.00; NFI = 0.947; RMSEA = 0.000).

**Figure 2:**
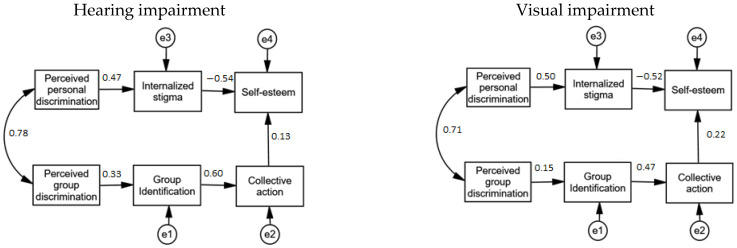
Multigroup model.

The multigroup model (Figure 2) also showed an excellent fit to the data in the unconstrained model (χ2 (18) = 18.10, *p* = 0.449; CFI = 1.00; NFI = 0.995; RMSEA = 0.005). This indicates that the patterns of fixed and non-fixed parameters in the research model were identical for the hearing and visual disabilities samples. The parameter estimates for both groups are shown in Figure 2. In the hearing-impaired group, all paths were significant except one: collective action does not predict self-esteem (β = 0.13, *p* = 0.13). In the visual impairment group, there was also only one non-significant path: perceived group discrimination does not predict group identification (β = 0.15, *p* = 0.15).

In the constrained model, the fit also was adequate (χ2 (23) = 24.75, *p* = 0.364; CFI = 0.995; NFI = 0.954; RMSEA = 0.020). A chi-square difference test showed that there were no significant differences in the model fit between the constrained model (constraining the structural weights parameters in the model to be equal across the two subgroups) and the unconstrained model (Δχ2 = 6.65; Δdf = 5, *p* > 0.05; ΔCFI = 0.005). Therefore, we can conclude that the parameter coefficients do not differ for hearing and visual disabilities.

### 3.3. Mediation Analysis

Table 3 shows the estimates of the direct and indirect effects regarding the interrelationships between the variables on the full, unconstrained SEM model. There was an indirect relationship between individual discrimination and self-esteem mediated by internalized stigma in both groups. To analyze the type of mediation, we restricted the paths from perceived individual discrimination to internalized stigma and from internalized stigma to self-esteem to 0 in the direct model in both groups. In this case, the direct relationship between perceived individual discrimination and self-esteem was significant for both the hearing impairment β = −0.276 (*p* = 0.000) and visual impairment β = −0.171 (*p* = 0.000) groups, and it decreased to β = −0.059 (*p* = 0.000) in the hearing impairment group and to β = −0.119 (*p* = 0.000) in the visual impairment group when internalized stigma was introduced into the model (full mediation). Thus, the bootstrapping results revealed the mediating effect of internalized stigma in the hearing impairment (β = −0.256, *p* = 0.000; 95% CI: −0.380, −0.144) and visual impairment (β = −0.260, *p* = 0.000; 95% CI: −0.399, −0.146) groups.

There was no mediating effect in the relationship between internalized stigma and self-esteem by group identification and collective action because the direct effect between group discrimination and self-esteem was not significant for either the hearing impairment (β = 0.131, *p* = 0.181) or visual impairment (β = 0.126, *p* = 0.214) groups, so there was no point in checking the mediating effect of both variables.

## 4. Discussion

People with hearing and visual impairment suffer from stigma and discrimination in many aspects of their daily lives. However, as far as we know, to date, there have been no studies analyzing the effect of this discrimination on the well-being of people with sensorial disabilities. The aim of this study was to test the relationship between perceived (personal and group) discrimination and self-esteem in a sample of Spanish people with hearing or visual impairments. We proposed a multigroup model (Figure 1) in which perceived personal discrimination and perceived group discrimination are related to people’s self-esteem through different paths. Personal perceived discrimination is related to self-esteem through an individual path (increasing internalized stigma), and group perceived discrimination is related to self-esteem through a social path that increases group identification and, in turn, collective action intentions. This model showed a good fit with the data for the multigroup model.

Our results show that, both in the case of people with hearing and visual impairments, perceived personal discrimination is strongly associated with internalized stigma, which, in turn, has a negative correlation with self-esteem. The association between perceived discrimination and internalized stigma has been found in several studies conducted with devaluated groups, mainly people with HIV and people with mental illnesses [25,26], but this is the first time that it has been studied in people with sensorial disabilities. The results are also consistent with the notion that internalized stigma plays a mediating role in the association between perceived personal discrimination and self-esteem. This means that when people with hearing and visual impairments feel discriminated against, they tend to internalize the stigma or to assume the negative opinion of the society about them, and this is negative for their self-esteem. Regarding people with visual impairments, we could not find a significant direct correlation between individual perceived discrimination and self-esteem. In this case, the internalization of stigma plays a mediational role: individual perceived discrimination is only harmful for the self-esteem of people with visual impairments when the negative social stereotype is endorsed (or internalized) by the individual. 

The pathway from group discrimination to self-esteem is not as clearly supported by the data. In the case of people with hearing impairments, we observed that perceived group discrimination leads to group identification and that group identification leads to collective action. This relationship might be related to the existence of a strong deaf identity and culture, which has been widely reported in the literature [36,37]. However, in our sample, collective intention does not strongly influence participants’ self-esteem. 

In contrast, in the case of people with visual impairments, perceived group discrimination does not lead to group identification, so this path does not work in order to explain the association between perceived group discrimination and self-esteem.

In sum, the proposed multigroup model for people with hearing and visual impairments shows that the individual path—that is, the association among personal perceived discrimination, internalized stigma, and self-esteem—is stronger than the social path. In this case, contrary to what happens with other stigmatized groups (for example, groups based on racial categorization), group discrimination (in the case of people with visual impairments) does not lead to group identification, and collective action intention has a low degree of relationship to the participants’ self-esteem for both kinds of disabilities. More research is needed to study the characteristics of group identity in people with sensorial impairments. Factors such as how long the individual has lived with the impairment and the severity and progression of the disability may influence the willingness to self-identify with an in-group based on the disability. However, this lack of identification has negative aspects for stigmatized people because it prevents them from taking advantage of the benefits of group membership [28,56,57].

The present study has some limitations. On the one hand, the study used a cross-sectional design. Thus, the relationships between the variables in the model should be tested in a longitudinal or experimental design in order to assert causality. On the other hand, the model included only one measure of well-being: self-esteem. 

In future research it would be interesting to consider other criterion variables in the model, such as participants’ positive and negative affect or participants’ psychological well-being. It is also necessary to explore the characteristics of the social identity in people with hearing or visual impairments in greater depth, and the role that the identity may play in order to cope with the effects of perceived discrimination. Moreover, the characteristics of the disability, such as its duration or its severity, may also be factors important for explaining the effects of discrimination and ways to cope with them [58].

## 5. Conclusions

This research is relevant from both theoretical and practical perspectives. From the theoretical point of view, our study explores, for the first time, the association between perceived discrimination and self-esteem in people with hearing and visual impairments. From a practical perspective, our research also has important implications for the development of intervention strategies. The results suggest that interventions aimed at improving the quality of life of people with sensorial impairments should focus on preventing the internalization of stigma in order to protect their self-esteem. Needless to say, the roots of the problem of the stigma concerning people with sensorial disabilities are external to them, and interventions on the general population level and the societal level are necessary, but interventions in stigmatized populations have been shown to improve the well-being of members of several stigmatized groups. 

## Figures and Tables

**Table 1:** Descriptive statistics and Pearson correlation coefficients for the sample of people with hearing impairments.

Variables	M	SD	2	3	4	5	6
Perceived personal discrimination	2.38	0.76	0.79 **	0.47 **	0.24 *	0.02	−0.21 *
2. Perceived group discrimination	2.59	0.70		0.44 **	0.33 **	0.09	−0.13
3. Internalized stigma	2.20	0.62			0.19	0.11	−0.53 **
4. Group identification	2.99	0.66				0.60 **	0.08
5. Collective action	3.25	0.69					0.07
6. Self-esteem	3.15	0.51					

Note. Scores range from 1 to 4. * *p* < 0.05. ** *p* < 0.01.

**Table 2:** Descriptive statistics and Pearson correlation coefficients for the sample of people with visual impairments.

Variables	M	SD	2	3	4	5	6
Perceived personal discrimination	2.03	0.72	0.71 **	0.50 **	0.09	−0.02	−0.17
2. Perceived group discrimination	2.27	0.66		0.34 **	0.15	0.08	−0.13
3. Internalized stigma	1.96	0.61			0.05	−0.19	−0.55 **
4. Group identification	2.87	0.72				0.47 **	0.03
5. Collective action	3.36	0.60					0.31 **
6. Self-esteem	3.33	0.52					

Note. Scores ranged from 1 to 4. * *p* < 0.05. ** *p* < 0.01.

**Table 3:** Results of mediation analysis.

Mediation Analyses	Direct Beta without Mediator	Direct Beta with Mediator	Indirect Beta [CI]
Personal Discrimination → Internalized Stigma → Self-Esteem			
Hearing impairment	−0.276 *	0.059	−0.256 *** [−0.380–−0.144]
Visual impairment	−0.171 *	0.119	−0.260 *** [−0.399–−0.146]
Group Discrimination → Group Identification and Collective Action → Self-Esteem			
Hearing impairment	−131	−0.115	0.025 [−0.004–0.083]
Visual impairment	−0.126	0.03	0.015 [−0.003–0.055]

* *p* < 0.05. *** *p* < 0.001.

## Data Availability

The data presented in this study are available on request from the corresponding author.
